# Investigating the Feasibility of Brief Compassion Focused Therapy in Individuals in Treatment for Opioid Use Disorder

**DOI:** 10.1177/1178221819836726

**Published:** 2019-04-02

**Authors:** Molly Carlyle, Helen Rockliff, Rachel Edwards, Crina Ene, Anke Karl, Beth Marsh, Lucie Hartley, Celia JA Morgan

**Affiliations:** 1 Psychopharmacology and Addiction Research Centre (PARC), College of Life and Environmental Science, University of Exeter, Exeter, UK; 2 Together Drug and Alcohol Services, Exeter, UK; 3 Clinical Psychopharmacology Unit, Research Department of Clinical, Educational and Health Psychology, University College London, London, UK

**Keywords:** self-compassion, opioid, addiction, feasibility, pilot intervention, craving

## Abstract

Opioid use disorder (OUD) is reaching epidemic proportions worldwide, and is notoriously difficult to treat. Compassion focused therapy (CFT) has emerged as therapeutic tool for treating individuals exhibiting high levels of self-criticism and low self-esteem, both of which are common in OUD. Until now, however, there had been no research investigating this therapy in patients with OUD. Before running a premature clinical trial, it is important to fully assess the feasibility and acceptability of this treatment in this group of individuals. We aimed to assess the feasibility of CFT treatment in individuals with OUD in a short group intervention, which was co-created by the research team, service users and a local drugs service. The intervention involved three 2-hour sessions held over 3 weeks, where participants engaged in compassion-orientated psychoeducation and self-compassionate exercises. Individuals were randomly assigned to either the CFT group (n = 15), the active control (relaxation) group (n = 12) or the waitlist control group (n = 11). Of 103 individuals approached, 45% attended a baseline visit suggesting the treatment was acceptable to this group. A relatively low attrition rate across the 3 groups was found for CFT (21.1%), with no difference in drop-out between the groups. Qualitative analysis of interviews with participants identified a desire for more sessions. Compassion focused therapy was thus feasible and well-tolerated in those with OUD, and a further trial to evaluate any clinical differences may be warranted.

## Introduction

Opioid use disorder (OUD) is responsible for most illicit drug-related deaths worldwide,^1^ which have recently reached epidemic levels.^2,3^ Rates of psychological trauma, particularly those that have occurred in childhood, are disproportionately high among those living with substance use disorders.^4,5^ Such experiences include inconsistent parental responsiveness, lack of affection, neglect, bullying and abuse,^6^ all of which are vulnerability factors to later developing a substance use disorder.^7^ Adverse experiences in early life can interfere with the adaptive development of emotion regulation which is typically acquired in childhood and adolescence.^8^ It has been suggested that individuals may use analgesic drugs such as opioids as a form of ‘emotional numbing’ to deal with unpleasant emotional states when the ability to self-regulate emotions has not been nurtured in childhood.^8^ Unpleasant emotional states that persist following experiences of early adversity can include high levels of self-criticism, guilt and shame,^9-11^ all of which are frequently reported among those living with addictions.^12,13^

Compassion has been conceptualised as sensitivity to suffering in self and others with a commitment to try to alleviate and prevent it.^14-16^ Self-compassion has been shown as protective for mental health and well-being.^17^ It is positively associated with improved emotion regulation abilities, and mediates the relationship between childhood trauma and later emotional dysregulation.^18^ Self-compassion is also related to reduced drug and alcohol use. People with severe alcohol use disorder who rate higher in self-compassion have better mental health, longer abstinence and lower levels of negative emotional states such as stress, depression, anxiety^19^ and self-criticism.^20^ Importantly, self-compassion was recently shown as inversely related to risk of developing a substance use disorder, potentially indicating its protective involvement in reducing problematic drug use and demonstrating its therapeutic value.^21^ Treatments aimed at fostering self-compassion have already proven highly successful in the treatment of mental health problems, particularly in people who express high levels of shame and guilt^22^ and have histories of trauma.^23^ Thus, it seems plausible that an intervention focused on compassion may also help those living with OUD. One issue with such treatments, however, is a resistance to engage in self-compassion from individuals for whom such experiences are alien and often aversive.^24^ Therefore, this study set out to examine the feasibility and acceptability of a brief, 3-session intervention developed to foster compassion in individuals in treatment for OUD, currently maintained on opioid substitution medication.

Third-wave psychological therapies are being increasingly used for treatment-resistant conditions, such as Acceptance and Commitment Therapy (ACT) or Mindfulness-based Cognitive Therapy (MBCT). However, the difference between these therapies and compassion focused therapy (CFT) is the inclusion of an explicit element that fosters the fundamental ability to self-soothe by developing the courage to engage with difficult emotions. Such self-compassion in those with OUD may be particularly beneficial, as external substances may be used as a compensatory mechanism to the absence of this process^25^ or to evoke positive emotions that have not previously been experienced due to childhood adversity. Compassion focused therapy focuses on helping people understand that the human brain has evolved in a way that makes it susceptible to rumination, negativity bias and self-critical self-monitoring.^26,27^ In therapy, individuals learn to shift attention from shaming the self for these difficulties to how to work with them compassionately.^28^ This is particularly relevant in opioid use where users feel large amounts of shame both currently around their drug use and historically due to trauma. Physiologically, recent research has demonstrated that CFT leads to improvements in heart rate variability.^29^ Heart rate variability is a measure of emotional regulation, and therefore, enhancing emotional regulation is key to recovery, as users most frequently report taking opioids to manage difficult emotions. The current intervention was brief to investigate whether it could exert a high impact and cost-effective means of treating OUD in drug services. The intervention drew on the principles of CFT: a novel treatment formulated to increase levels of self-compassion.^30,31^ This study merged principles from 2 leading models in the literature: one of which is rooted in evolution and attachment^31^ while the other is informed by eastern meditative traditions.^15^

## Method

### Participants and design

This study was a mixed-methods design. Participants were allocated to either CFT, active control (relaxation training) or waitlist control. Compassion focused therapy and the active control group were randomised, and participants were blind to whether they were in the active treatment or active control.

The final sample consisted of 38 participants (24 men; 14 women) aged 22–62 years (M = 39.95, SD = 10.44) currently in treatment with drug services and prescribed a daily opioid substitute medication (OSM: methadone; buprenorphine). All participants had a diagnosis of OUD.

The study was advertised as a ‘stress reduction skills course’ to reduce preexisting expectations about what the groups involved. Data from the waitlist control were collected subsequent to the first 2 groups, and this group was offered treatment following their participation. Inclusion criteria were above 18 years of age, fluent English speakers and currently taking an OSM. Participants were excluded if they had learning difficulties or neurological impairment, or were illiterate. The study was approved by the institutional university ethics committee.

### Intervention development

The CFT sessions involved a mixture of psychoeducation with experiential exercises (see Table 1 for details of the intervention) and were co-created by a team of psychologists, drug workers from the local drug service and service users in recovery. The brief format was judged as acceptable by users and key workers, and as having a good chance of treatment adherence to maximise retention and engagement.

**Table 1.** Content of sessions for active treatment and control groups.

	CFT treatment	Relaxation training
Session 1		
Psychoeducation	Building the foundations for understanding compassion: • ‘Tricky Brain Loops’^a^: Understanding the brain as a product of evolution, and that human cognitive capacities for imagination, planning, and ruminating (this ‘newer brain’) can become easily hijacked by our ‘emotional brain’, where our capacity to reflect on negative events can activate negative and fearful emotions. • The Social Brain^a^: Understanding that the development of social relationships is important for functioning, and we require socially safe connections with others to thrive. • The Three System Model: Understanding behaviour is driven by 3 emotion regulation systems: (1) a threat-focused protection system (such as the fight or flight), (2) a motivational drive system for rewards and achieving goals, and (3) an affiliative system for feeling safe and self-soothed. If the affiliative system is not nurtured through attachment and care from others, the 2 other systems can dominate. Attempts to highlight that these brain loops are not our fault, but it is us that suffer if we do not take responsibility for them. • ‘Two Worlds’: Human brains and bodies respond to both an external world and an internal world of thoughts/self-talk. The role of self-compassion and self-criticism in responding to difficulties and distress, and human suffering.	• Fight or flight response: Understanding that the brain has evolved to respond to stress automatically, but this response can be problematic in modern day society. • Using relaxation to combat stress: Discussing the benefits of relaxation in reducing the negative outcomes caused by stress. • Introducing relaxation with breathing: Discuss how controlling breathing can help us relax. • Combating stress with visualisation: Discuss the power and benefits of visualisations.
Exercises	• Mindful eating: Mindfulness is fundamental for compassion and thus was practised within the sessions. This exercise involved eating food with more sensory awareness; taking care to notice the smell, feel, taste and texture. • Monitoring the 3 channels (thoughts, feelings, body, and behaviours): This was a written/discussion exercise aimed at fostering awareness. Participants were asked to imagine a situation, and the thoughts, feelings, and behaviours associated with it. • Compassionate body scan: A guided imagery exercise where you consider each part of your body and show gratitude to it, and was the first exercise that explicitly focused on compassion.	• Belly breathing: A simple exercise that uses a deeper and slower form of breathing. • Rectangular breathing: The use of an external, rectangular object to guide our breathing pace (shorter inhales, longer exhales). • Guided beach visualisation: As well as using slow breathing, we are guided through a relaxing beach scene, with eyes closed.
Session 2		
Psychoeducation	Learning compassion: • Defining compassion^a,b^: Understanding and defining what compassion is, with reference to the evolutionary-attachment approach^a^, as well as from a Buddhist perspective in understanding suffering as part of the human condition^b^. • Common misconceptions: Understanding that compassion is not an overindulgence, a weakness, or demotivating^a,b^. Compassion can be misinterpreted in these ways, but it is a strength that promotes motivation and health. • Different components of compassion: Discussing that compassion is made up of 3 components: (1) self-kindness, (2) common humanity, and (3) mindfulness^b^ that can all be fostered to develop a compassionate mind.	• Combining breathing techniques and visualisation • How muscles hold tension: muscles can hold a lot of tension due to stress, which can be painful and unhealthy.
Exercises	• Considering ‘how do I treat a friend versus myself?’: This was a written exercise where participants were asked to imagine the misfortune of a friend, and write what they would say to them. This exercise was used to highlight the disparity between the compassion we show for others versus the compassion we show to ourselves, and that we can be highly critical to ourselves in times of distress. • Write a compassionate letter to the self: This was a written exercise where participants were then asked to imagine a time of their own misfortune and suffering, and to write a compassionate letter to themselves.	• Colour breathing (breathing colours that make us feel relaxed) • Progressive muscle relaxation (deliberately tensing and relaxing each muscle group)
Session 3		
Psychoeducation	Difficulties with training compassion: • Discussing fears surrounding compassion (‘compassion blockers’): Understand why compassion could be difficult, and how it can bring up painful past memories where compassion has been sought but unfulfilled. Fears of compassion fear of feeling safe can arise due to trauma in childhood, possibly via classical conditioning between abuse and family members or the home (people and a place that are supposed to feel safe)^a^. • Undoing these ‘blockers’: Comparing compassion as an under practised skill that is difficult at first but can be trained by using techniques and exercises that were practised within the sessions	Combining techniques that were learned in past sessions: Breathing, visualisations, and muscle relaxation can be used together.
Exercises	• Building a compassionate self: This was an auditory exercise aimed at developing the ability for participants to feel and behave compassionately to others and themselves. • An ideal compassionate self: This was another auditory exercise where participants were asked to imagine warm, supporting and caring emotions. This was done to try and enhance recognising these emotions. • Soothing rhythm breathing: A breathing exercise aimed at nurturing compassion by soothing the mind	Autogenic relaxation (imagining muscles as feeling heavy or warm)
Between session resources		
Mp3	Recordings of the exercises given in each session were recorded on an Mp3 and given to participants to practice in their own time, and were advised to use between sessions and in times of distress	
Keychain booklets	An attractively designed summary of the psychoeducation from each session in the form of a booklet was given to participants to take home. This enabled them to cover any information from the sessions in their own time.	

Abbreviation: CFT, compassion focused therapy.

a Informed by Paul Gilbert’s model of compassion.^31^

b Informed by Kristin Neff’s model of self-compassion.^32^

The relaxation training group emulated the experience of those in the active treatment group as closely as possible by containing a similar weighting of psychoeducation and exercises.

### Measures

#### Feasibility

Feasibility was assessed by assessing the percentage of individuals agreeing to take part in the study (success criteria: 60%), completing the baseline measures (success criteria: 70%) and completing follow-up measures (success criteria: 50%).

#### Measures

We included quantitative measures to be able to assess the feasibility of using such measures in a full trial. Additional measures were included to pilot the feasibility of the design in a larger trial which were questions around opioid prescription and are reported elsewhere.

#### Obsessive-Compulsive Drug Use Scale (OCDUS)

This assessed opioid drug craving, with 3 subscales – thoughts and interference, desire and control, and resistance to thoughts and intention^33^ – and the 3 subscales had good reliability when implemented before and after the intervention (thoughts and interference – before, after: α = .785, α = .788; desire and control – before, after: α = .914, α = .898; resistance to thoughts – before, after: α = .874, α = .809).

#### Depression Anxiety Stress Scale (DASS)

This scale measured levels of depression, anxiety and stress over the past week, and each subscale was shown to have high reliability when it was implemented both before and after the intervention (depression – before, after: α = .922, α = .887; anxiety – before, after: α = .816, α = .879; stress – before, after: α = .914, α = .930).^41^

#### The Forms of Self-criticising/Attacking and Self-reassuring Scale (FSCRS)

This measure assessed self-relating via self-criticism and self-reassurance/compassion. Subscales include feelings of self-inadequacy, ability to self-reassure and self-hate.^34^ Each subscale was shown to have reasonable to high reliability when it was implemented both before and after the intervention (self-inadequacy – before, after: α = .891, α = .874; self-reassurance – before, after: α = .822, α = .852; self-hate – before, after: α = .778, α = .858).

### Procedure

**Figure 1.**
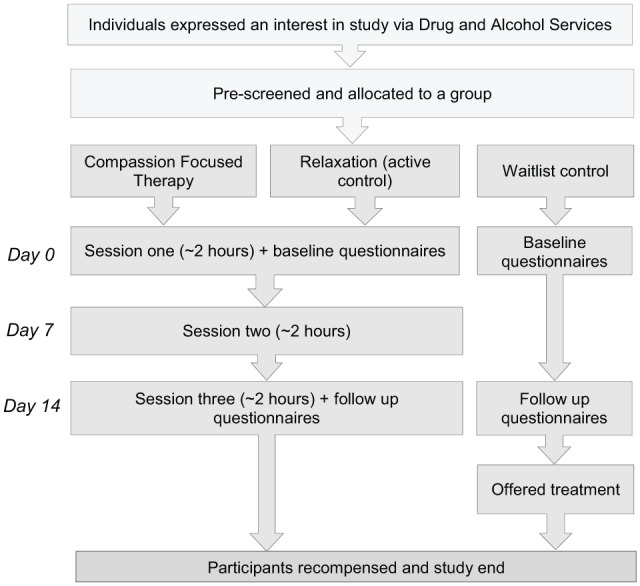
The study procedure.

Both the experimental and active control group attended three 2-hour sessions held over 3 consecutive weeks (Figure 1). The waitlist control group filled out the baseline measures, and repeated these on the third week for follow-up.

Prior to participating, participants were contacted for screening. This involved a brief assessment of participant’s drug use history and their OSM. Once screened, participants were allocated to either the CFT, relaxation or waitlist control group.

On arrival for session 1, participants provided informed consent and were then asked to complete the self-report baseline measures, which were completed again at the end of session 3. Between sessions, participants in the active groups were asked to engage in practical activities related to the session content by listening to guided recordings on an MP3 device and reading a short booklet. After the final session, we asked the group to feedback their views on their experiences, and in a semi-structured interview, these responses were recorded and later transcribed. On completion of the study, participants were reimbursed for their time with a voucher.

### Statistical analyses

#### Quantitative data

Data were analysed using the Statistical Package for Social Sciences (SPSS), version 23. Data were checked for normality and homogeneity. The primary outcome was feasibility. An exploratory analysis was conducted of the depression and anxiety and craving data with 3 × 2 repeated-measures analysis of variance (ANOVA) and following up large or medium effect sizes with mean differences and confidence intervals (CIs) in line with recent recommendations for reporting pilot studies.^35,36^

#### Qualitative analysis

Interview data from session 3 were transcribed and entered into NVivo software (11.1, QSR International). A thematic analysis was conducted of the interview data collected from the CFT group; data were coded on themes relating to the acceptability and effects of the intervention by 2 independent raters (HR and CM). A single coding frame was designed which included perceived benefits of the intervention and comments on acceptability and feasibility. Two researchers (HR and CM) then went through the coding frame to check for agreement. Each narrative was coded according to the appropriate coding frame (HR) with inter-rater reliability checked by random selection of 4 interviews (CM) and reached high agreement (>95%). Any discrepancies were resolved through discussion or, if an agreement could not be met or it was not clear what the participant was trying to get across, items were coded as ‘ambiguous’ (<0.02% items coded as ambiguous). This procedure produced 12 codes (not reported here). These codes were categorised into 4 thematic headings.

## Results

### Demographics

The treatment groups appeared matched in age, sex, years in education and indices of past illicit opioid use (see Table 2).

**Table 2.** Participant demographics between treatment conditions (means and standard deviations).

	Compassion focused therapy (n = 15)	Relaxation (n = 12)	Waitlist control (n = 11)
Age	41.07 (12.70)	43.33 (8.27)	34.82 (7.94)
Sex (n = men; women)	11; 4	7; 5	6; 5
Years in education (mean rank)	11.77 (2.24)	12.42 (2.23)	11.73 (0.79)
History of mental health problems	12	6	9
Opioid prescription type (n = methadone; buprenorphine; morphine)	8, 6, 0	11, 1, 0	8, 2, 1
Opioid prescription dose in mg, /d			
Methadone	55.63 (60.62)	42.36 (22.54)	51.25 (6.41)
Buprenorphine	10.00 (4.56)	16.00 (0.00)	14.00 (2.83)
Morphine	560.00 (0.00)	-	-
Length taken opioid prescription, y	9.79 (7.70)	6.80 (4.66)	7.39 (5.84)
Last use of illicit opioids (excluding prescription), d (mean rank)	21.46	16.33	16.95
Time since first started using opioids, y	17.41 (12.13)	20.25 (6.47)	14.50 (8.57)
Peak use of opioids following onset, mo	46.46 (29.98)	60.38 (34.56)	24.72 (34.17)
Money spent on opioids at peak use, £ pounds sterling /d	175.80 (144.52)	105.50 (101.80)	88.80 (77.61)
Currently using other illicit substances	7	8	8

Means and standard deviations are provided where data are parametric, otherwise the mean rank is given in cases that are non-parametric. Total counts are given for categorical variables.

### Treatment uptake and adherence

In all, 103 individuals were interested and contacted by the research team, where 69 (66.99%) of these individuals agreed to take part in the study. Of these, 47 (45% of all approached; 68.12% of those who agreed to take part) attended the first session (baseline), and 38 (80.85% of those attending baseline visit) continued to complete the full study and completed all measures – CFT: n = 15 (n = 4 discontinued treatment); relaxation: n = 12 (n = 1 discontinued treatment), and waitlist: n = 11 (n = 4 lost to follow-up) – with no significant differences in number of drop-outs between the groups (χ^2^= 2.62, n = 38, *P* = .270).

### Quantitative analysis

#### Craving

**Figure 2.**
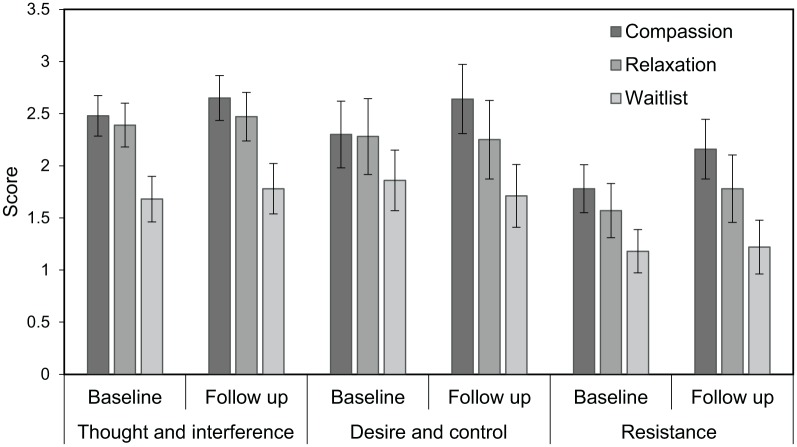
Scores on craving subscales between each group. There were medium effect sizes for `desire and control’, and `resistance’ subscales which were followed up by mean differences and confidence intervals. These indicated that the compassion group were experiencing a larger change in scores from baseline to follow up than the waitlist control group, where scores were higher at follow up.

A 3 × 2 mixed repeated measures ANOVA ‘thoughts and intentions around opioid use’ showed a main effect of group, F_2,34_ = 4.88, *P* = .014, η^2^ = 0.22, associated with a large effect size, where pairwise comparisons revealed that the waitlist control group had lower scores overall than both the compassion (*P* = .005) and relaxation (*P* = .021) groups (Figure 2). There were no differences in scores from baseline to follow–up, F_1,34_ = 1.26, *P* = .269, η^2^ = 0.04, and no interaction between group and time, F_2,34_ = 0.06, *P* = .939, η^2^ < 0.01.

For ‘desire and control over opioid use’, there were no main effects of group, F_2,24_ = 1.50, *P* = .242, η^2^ = 0.11; time, F_1,24_ = 0.09, *P* = .769, η^2^ < 0.01; and no interaction, F_2,34_ = 1.37, *P* = .273, η^2^ = 0.10, although the latter was a medium effect size (Figure 2). Mean difference scores between the compassion and relaxation group were 0.22 (80% CI: –0.40-0.83), and compassion group and the waitlist group were 0.71 (80% CI: 0.15-1.25).

For ‘resistance to thoughts and intentions surrounding opioid use’, there was a difference in scores between baseline and follow-up measures that approached significance, F_1,24_ = 3.43, *P* = .077, η^2^ = 0.12 (Figure 2). There was also a trend for a difference in scores between groups, F_2,24_ = 2.91, *P* = .074, η^2^ = 0.20. The interaction between group and time, F_2,24_ = 0.82, *P* = .452, η^2^ = 0.06, were associated with a medium effect size (Figure 2). Mean difference scores between the compassion and relaxation groups were 0.29 (80% CI: –0.18-0.77) and between the compassion and waitlist group were 0.77 (80% CI: 0.34-1.19).

#### Depression, anxiety, and stress

A 3 × 2 repeated measures ANOVA on depression scores found a main effect of time, F_1,35_ = 6.83, *P* = .013, η^2^ = 0.16, where there was an overall decrease in scores from baseline to follow-up, associated with a large effect size (Table 3). There were no differences between groups, F_2,35_ = 0.97, *P* = .389, η^2^ = 0.05, and no interaction, F_2,35_ = 0.01, *P* = .995, η^2^ < 0.01.

**Table 3.** Means and standard deviations for scores on anxiety, depression and stress scales.

	Compassion	Relaxation	Waitlist
Depression			
Baseline	12.13 (5.32)	12.08 (6.96)	14.55 (4.80)
Follow-up	9.93 (5.32)	10.00 (6.35)	12.55 (4.41)
Anxiety			
Baseline	9.53 (3.91)	11.17 (6.90)	7.11 (5.69)
Follow-up	9.05 (5.59)	8.18 (5.38)	8.67 (6.34)
Stress			
Baseline	12.62 (5.25)	12.29 (7.49)	12.55 (4.16)
Follow-up	11.49 (6.01)	8.83 (6.13)	11.73 (5.37)

For anxiety, a similar 3 × 2 repeated-measures ANOVA found an interaction between treatment type and time that approached statistical significance, F_2,33_ = 3.08, *P* = .059, η^2^ = 0.15, and was associated with a large effect size. The mean difference scores between the compassion group with relaxation and waitlist control groups were −0.38 (80% CI: –3.01-2.25) and 1.40 (80% CI: –1.46-4.27), respectively. There were no main effects of group, F_2,33_ = 0.33, *P* = .723, η^2^ = 0.02, or time, F_1,33_ = 0.79, *P* = .381, η^2^ = 0.02.

There was a main effect of time on stress, F_1,35_ = 9.21, *P* = .005, η^2^ = 0.19, associated with a large effect size, where scores reduced from baseline to follow-up in all groups (Figure 1). There were no differences between groups, F_2,35_ = 0.31, *P* = .734, η^2^ = 0.01, or statistically significant interaction, F_2,35_ = 1.91, *P* = .163, η^2^ = 0.08; however, the interaction was associated with a medium effect size. The mean difference scores between the compassion group with relaxation and waitlist control groups were 1.49 (80% CI: –1.31-4.30) and −0.08 (80% CI: −2.96-2.80), respectively.

#### Self-criticism (FSCRS)

A 3 × 2 mixed ANOVA were conducted on subscales of self-criticism. There were no significant effects on self-hate or self-reassurance and all effect sizes were small. The analysis of scores for the self-inadequacy subscale found an interaction associated with a medium effect size, F_2,35_ = 0.77, *P* = .185, η^2^ = 0.09; however, mean difference scores and 80% CIs revealed no further differences between the compassion and relaxation or waitlist control group: 0.09 (80% CI: –0.58-0.40) and <0.01 (80% CI: –0.49-0.51), respectively.

### Qualitative analysis

From transcripts of the interviews that followed the end of the final session, a coding framework was developed. Four themes emerged from the analysis of the qualitative data. Three themes related to the effects of the intervention:

1. Better understanding of oneself and others

A number of participants in the compassion group (n = 7) reported as an outcome experiences that could be coded under the thematic heading of ‘*better understanding of oneself and others*’:

. . . Yeah, I mean like I used to get annoyed with people that would look for sympathy all the time. Now I can understand why they’re doing it, because they’re looking for that part of the brain that needs like filling up basically. (Participant 9)

2. Increased strength in the face of difficulty

Another emergent theme mentioned by a number of participants (n = 6) was categorised as ‘*increased strength in the face of difficulty*’. Participants reported that a positive impact of the CFT was on their ability to sit with discomfort and not to over-engage or feel the need to shut down or use to numb their emotions:

. . . I mean I come here and I have found a few new tools to help me. Well, I’m always going to be going through life and coming across stressful situations, I can’t keep running away or doing short cuts. (Participant 11)

3. Reducing self-criticism

Another one relating the effects of the intervention was in reducing self-criticism (n = 4) in participants, reporting that the intervention had given them some enhanced understanding of their own self-critical inner voices and an acceptance of their own imperfections: ‘It enables you not to be so harsh on yourself doesn’t it? Because you see that there’s reason for everything, so I can’t be perfect. It’s okay not to be perfect as well’ (Participant 13).

4. Intervention was too brief

The other theme emergent from many of the interviews following the final session was the need for more sessions (n = 4): ‘Personally I think they could have done with a bit more, because just basically you’re scratching the surface ain’t you and then it’s the end’.

## Discussion

The current study piloted a novel intervention aimed at fostering compassion in those with OUD. In relation to the primary aim of the study, a short-course of CFT in opioid drug users appears to be feasible in this population. The results of the study suggest that the intervention is feasible, and may warrant further investigation in a larger randomised control trial. Retention rates were high: of those who attended the first session, 81% continued to complete the full study. The attrition rate did not differ between groups. Attrition rates during CFT in other groups have been previously reported to be high in some cases, particularly among non-clinical groups.^24^ Treating oneself with compassion can feel alien, and has the potential to cause distressing emotional reactions in those with histories of abuse and neglect, thus causing individuals to discontinue treatment.^24^ Despite this, our study had particularly high retention for this population, with similar mindfulness-based interventions in opioid users reporting retention rates between 45% and 75%.^37^ As CFT had equally good retention as relaxation, it may be a promising approach for opioid users with trauma history.

Thematic analyses of interview data suggested positive effects from individuals in 3 areas: reducing self-criticism, facing negative emotions and a better understanding of themselves. The brief intervention approach was chosen following consultation with service users and workers given considerations about engagement with other psychotherapeutic groups, but the qualitative analysis suggested that a future study might consider a longer intervention, as several of the participants reported that they wanted more sessions. This, however, should be balanced against the good retention rates of the study that contrast to other similar yet longer interventions in the same population.^37^

The study was not powered to detect clinical change, and pilot and feasibility studies have been criticised for relying on inferential statistics and hypothesis testing when they are underpowered to detect clinically meaningful effects.^38^ It has therefore been suggested that examining effect sizes and reporting the mean differences and CIs can be more informative to identify whether the intervention is worth taking forward to a larger, randomised controlled trial.^36^ We performed exploratory analyses guided by these principles. We found that, on the OCDUS, the compassion group not only rated themselves as having higher desire for opioids following the intervention but also rated themselves as making more effort to resist the urge to use opioids, compared with the waitlist control group. These findings were in opposite directions, and may be treated with caution given the limited scope of this pilot study to address clinical questions. Tentatively, we might interpret these findings as reflecting the enhanced desire to use drugs as part of the difficult feelings that CFT may bring up in individuals, which might further suggest the need for a longer intervention. Theoretically, CFT helps individuals engage with feelings of disconnection and loneliness which underlie drug use, and therefore, an increase in craving for drugs in the early stages of therapy might be predicted as a function of enhanced awareness of these issues. Resistance may come as a function of enhanced urges to use, and any future study should assess actual drug use using objective measures to examine whether CFT produced increases in drug use in these groups. In our exploratory quantitative analysis, we also observed a reduction in depression across all groups in the study, likely either a function of regression to the mean or of being in a research study. Stress and anxiety appeared to reduce in the relaxation group compared with the compassion group, but there no differences between the compassion and waitlist controls. Relaxation is an effective therapy in the short term in addiction and this likely mediated these changes. Overall, these quantitative measures were feasible to use in this setting and a future adequately powered trial can assess the impact of CFT on these outcomes.

Although the study was not powered to detect clinical outcomes, the current project observed overall declines in depression and stress in all 3 groups. It is possible this may be a result of regression to the mean: where participants’ ratings on these scales are more extreme at baseline, and closer to the average at follow-up. The relaxation group performed equally well as the compassion group in depression and showed a greater reduction in anxiety, which is perhaps to be expected with an intervention like relaxation that targets anxiety and is effective over a short time frame. In a fully powered clinical trial, it would be interesting to see whether a longer compassion intervention would be able to replicate the enhanced outcomes following compassion compared with relaxation that have been observed in other clinical groups.^39^

One limitation of the study is its scope and small sample size. However, we have been able to assess the feasibility of this design, which was the primary aim of the study. Although therapists were supervised by a clinician who was experienced in CFT, another limitation was the absence of checks of fidelity to CFT treatment. Another limitation is that much of the content focused on psychoeducation around compassion and CFT generally, and might have further emphasised how the people involved might use compassionate practises to cope with their opioid use. Indeed, one suggestion of a future direction, rather than using compassionate body scans, would be to get users to imagine compassionate opioid practices where people imagine feeling craving for opioids and then switch to compassionate mind states and focus on imagining themselves resisting or reducing. One strength of this study is the use of 2 control groups, including an active comparator group to investigate the feasibility of such a trial design. The active groups were carefully matched in time and duration of sessions, and contained an equal balance of psychoeducation and experiential exercises. This design, if followed through to a fully powered trial, would enable us to understand the relative benefits of CFT in comparison with a similarly structured intervention, as well as the absolute benefits when compared with no treatment.^40^

## Conclusion

The use of CFT as an intervention for those with opioid addiction is feasible and has high adherence rates. The quantitative data do not suggest a positive effect of this brief intervention over relaxation therapy and there was some suggestion of a greater desire to use opioids at the end of the intervention which is concerning. Future work should explore whether this increase in craving is related to increased drug use, which may suggest a limited use for this therapy in this group. Qualitative reports from users, however, suggested some observed benefits and a desire for more sessions of the self-compassion intervention. Overall, these results indicate this new treatment to be feasible, and careful consideration should be given as to whether this should be systematically investigated in a higher powered, randomised control trial.
